# Seasonal Variation of Triacylglycerol Profile of Bovine Milk

**DOI:** 10.3390/metabo7020024

**Published:** 2017-06-02

**Authors:** Zhiqian Liu, Jianghui Wang, Benjamin G. Cocks, Simone Rochfort

**Affiliations:** 1 Biosciences Research, Agriculture Victoria, AgriBio, 5 Ring Road, Bundoora, Victoria 3083, Australia; Zhiqian.Liu@ecodev.vic.gov.au (Z.L.); Jianghui.Wang@ecodev.vic.gov.au (J.W.); Ben.Cocks@ecodev.vic.gov.au (B.G.C.); 2 School of Applied Systems Biology, La Trobe University, Bundoora, Victoria 3083, Australia

**Keywords:** milk, triacylglycerol profile, seasonal variation, liquid chromatography-mass spectrometry

## Abstract

Milk contains 3–6% of fat, of which the dominant component is triacylglycerol (TAG). Over 100 TAG groups can be readily detected in any non-enriched milk sample by LC-MS; most TAG groups contain several isomers (TAG molecules with different fatty acid composition), which cannot be fully resolved chromatographically by any single stationary phase. TAG profile of mature milk from 19 cows was surveyed in this study for eight consecutive months using RP-LC-Orbitrap MS. It was found that TAG profile of milk was not constant throughout the milking season and the seasonal pattern varied with TAG groups. The overall unsaturation level of TAG was stable from October 2013 to January 2014, decreased in February/March 2014 and then increased from April and peaked in May 2014. In addition to the seasonal fluctuation in TAG profile, the proportion of different isomeric species within a TAG group also changed substantially across seasons. However, the proportion of different positional isomers within a given TAG group does not seem to vary during the milking season. To our knowledge, this is the first report on the seasonal change of milk lipid at the TAG group and isomer level.

## 1. Introduction

Bovine milk contains 3–6% of fat, of which the dominant portion (about 98%) is in the form of triacylglycerols (TAG) located inside fat globules [1]. Polar lipids including phospholipids and sphingolipids represent 0.5–1% of total fat and are mainly found in the fat globule membrane [2].

It is well established that milk fat content and lipid composition vary with cow breed, animal diets, stage of lactation, and seasons [3,4,5,6,7]. However, most of the studies on lipid composition of milk focused on overall fatty acid profile, only a small number of reports can be found dealing with TAG structure [8,9]. Due to the fact that the distribution of fatty acids within TAG molecules are not random, the results obtained on global fatty acid composition of milk cannot be extrapolated to TAG profile. Consequently, how TAG profile and structure of bovine milk is influenced by genetic and environmental factors remains largely unknown.

It is widely recognized that TAG profile determines the physicochemical properties of milk fat [10,11,12,13], which are important for the processing and quality of dairy products. In addition, TAG structure could affect the absorption efficiency of fatty acids by infants [14,15]. Therefore, understanding the accumulation pattern of milk TAG is important for maximizing the functional and nutritional values of milk fat.

In this paper, we report a detailed study on seasonal variation of the most abundant TAG groups of milk collected in South Victoria, Australia. TAG profile was surveyed during a whole milking season (October 2013 to May 2014) for 19 Holstein cows.

## 2. Results

### 2.1. TAG Profile of Bovine Milk

**Figure 1:**
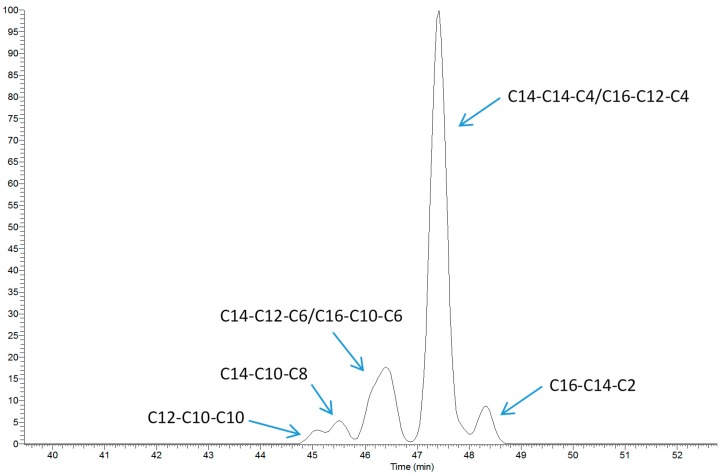
LC-MS profile (EIC) of TAG 32:0 isomers and their fatty acid composition.

If a TAG group is defined as a series of species having the same total acyl carbon number (CN) and the same number of total double bonds (DB), over 100 TAG groups were identified in non-enriched mature bovine milk using our LC-MS method. As expected, each TAG group contains a number of isomers of different fatty acid composition (fatty acid position on the glycerol backbone not taken into account); such isomers are regarded as different species. For example, TAG 32:0 is composed of seven isomers, which cannot be fully separated even with two C18 columns connected in series combined with a shallow gradient elution of 90 min (Figure 1). MS/MS scanning data allowed us to determine the fatty acid composition of the chromatographically resolved and non-resolved isomers [16], but reliable quantification of each isomer is not currently feasible for most TAG groups using one-dimensional LC [15,17]. Consequently, our survey on seasonal variation of TAG profile was conducted at the group level using a short LC method, which allowed all main isomers to be eluted as a single peak.

**Figure 2:**
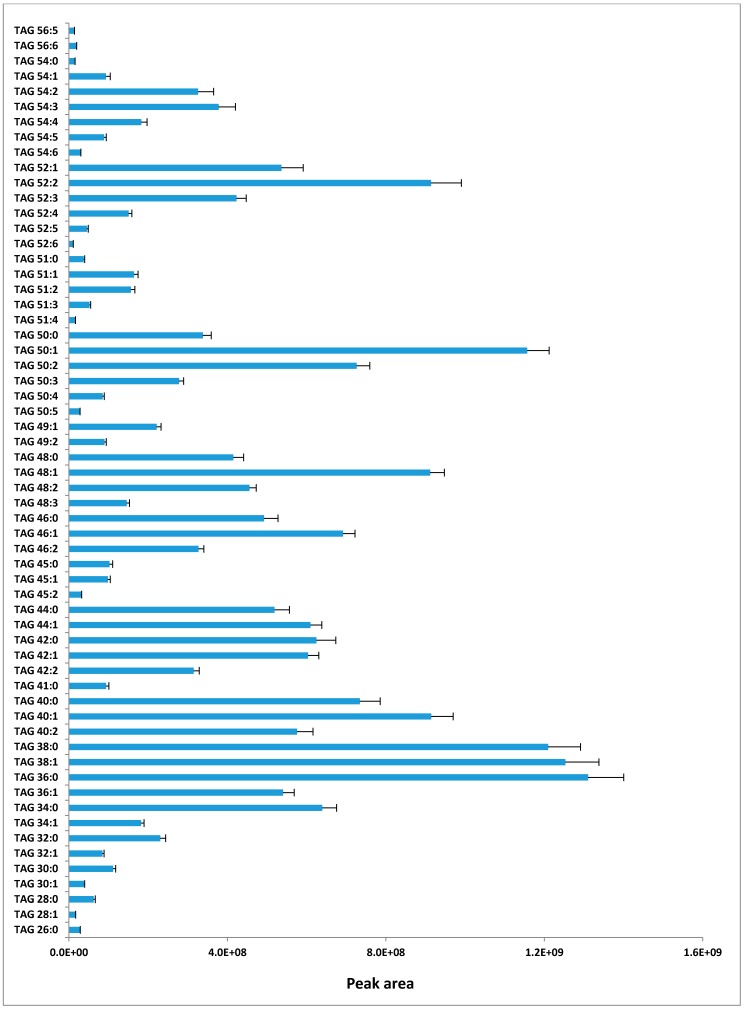
TAG groups surveyed and their relative abundance in milk. Each column is the mean value of 19 cows; error bars are standard errors.

The 60 most abundant TAG groups of milk were selected for detailed analysis; their identity and relative abundance (as estimated by the average peak area of all samples) are shown in Figure 2. These 60 TAG groups contain 26–56 CN and 0–6 DB, and display a multimodal distribution.

### 2.2. Correlation between TAG Groups and Total Fat Content

**Figure 3:**
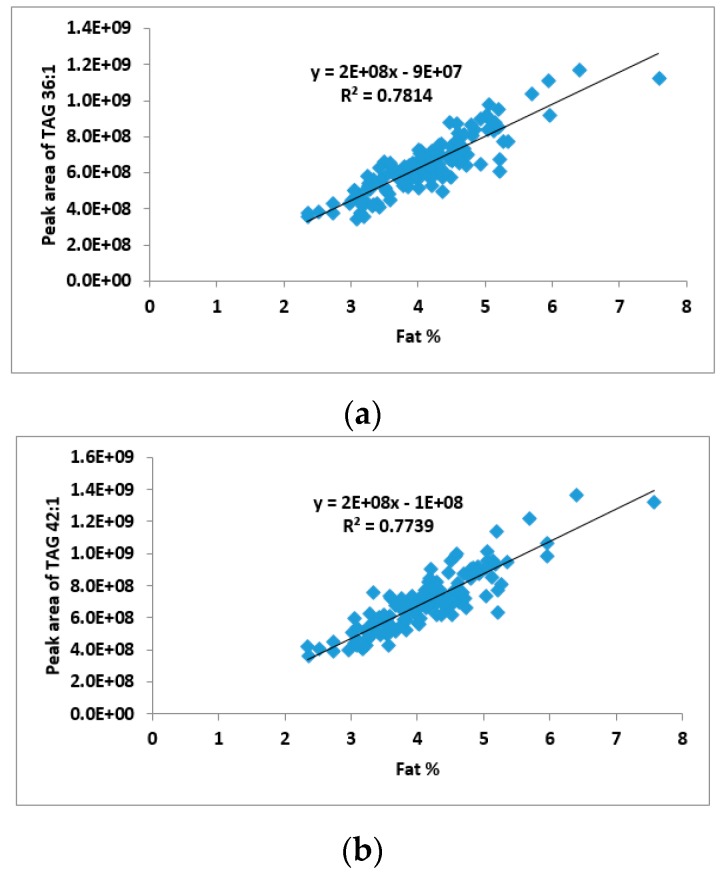
Correlation between two TAG groups and total fat content (*n* = 141). (**a**) Correlation between TAG 36:1 and total fat content; (**b**) correlation between TAG 42:1 and total fat content.

A total of 141 samples were collected from 19 cows during the eight-month milking season; the abundance of the 60 TAG groups in these samples was measured by LC-MS. A pairwise correlation analysis was conducted between the abundance of each of the 60 TAG groups and the total fat %, which was determined in parallel using an infrared-based technique [18]. All of the TAG groups positively correlated with the total fat content, but the correlation coefficient varied greatly across the TAG groups (Table S1, Supplementary Materials). The strongest correlation (R^2^ > 0.75) was observed between the total fat % and the abundance of both TAG 36:1 and TAG 42:1, suggesting that the abundance of these two TAG groups could be used to predict the total fat content of a milk sample (Figure 3).

### 2.3. Seasonal Variation of Milk TAG Profile

Due to the fluctuation of total fat content during the milking season, using the abundance value of each TAG group to assess the seasonal variation of TAG profile of milk is not appropriate. As a result, the percentage (or proportion) of each TAG group (calculated based on their peak area) was used to reveal the seasonal variation pattern of TAG profile.

**Figure 4:**
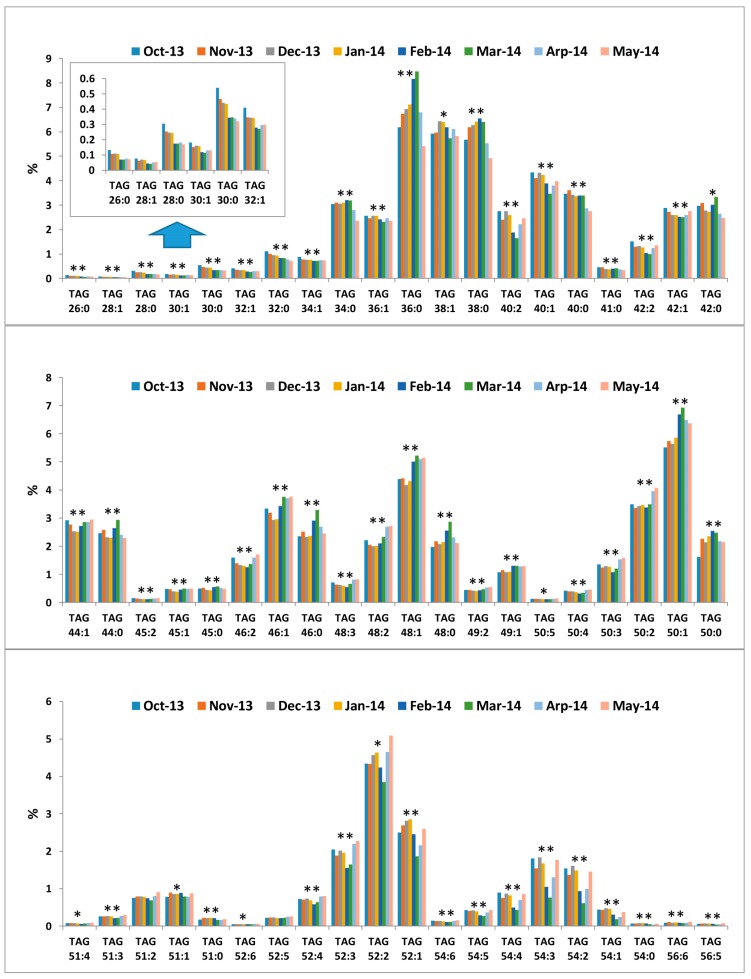
Seasonal variation of TAG profile. Each column is the mean value of 15–19 cows. Statistical difference across seasons is shown by ** (*p* < 0.01) and * (*p* < 0.05).

Overall, a significant seasonal variation was observed for the majority of the TAG groups surveyed (Table S2, Supplementary Materials), but the seasonal pattern appears to be complex and varies greatly between TAG groups (Figure 4). The following observations can be made:

(1) Not all TAG groups showed a clear seasonal change. For example, the proportion of TAG 36:1, TAG 38:1, and TAG 51:1 was relatively constant throughout the eight-month milking season (Figure 4).

(2) Some TAG groups showed a steady decrease over the milking season, so the lowest percentage was observed in April/May. TAG 26:0, TAG 28:0, TAG 30:0, and TAG 32:0 belong to this category (Figure 4).

(3) The proportion of some TAG groups did not change much for the first four months, but started to increase in February and peaked in March before declining again in April and in May. TAG 36:0, TAG 46:0, TAG 48:0, and TAG 50:1 followed such a seasonal changing pattern (Figure 4).

(4) The proportion of some TAG groups especially di-and poly-unsaturated TAG groups did not change much for the first four months, but started to decrease in February and March before increasing again in April and in May. TAG 40:2, TAG 52:3, TAG 52:2, TAG 52:1, TAG 54:5, TAG 54:4, TAG 54:3, TAG 54:2, and TAG 54:1 displayed such a seasonal variation pattern (Figure 4).

**Figure 5:**
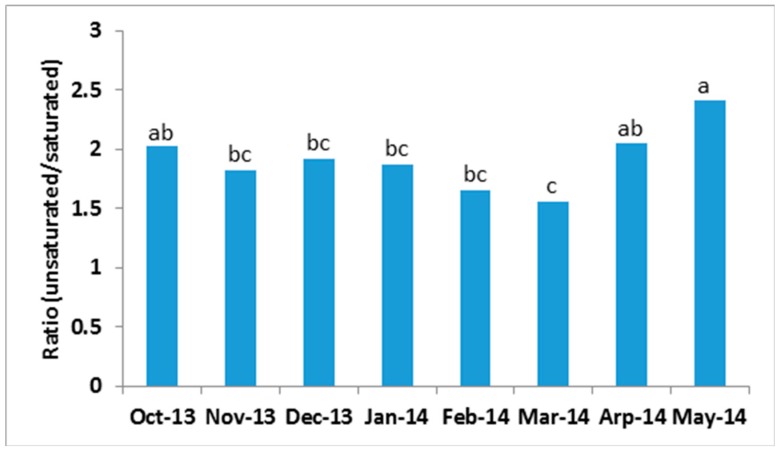
Seasonal change in ratio between unsaturated and saturated TAG. Each column is the mean value of 15–19 cows; columns with different letters are significantly different (*p* < 0.05).

If we calculate the ratio between unsaturated and saturated TAG, then we can see that this ratio remained relatively constant for the first four months, then started to decrease in February and reached a lowest value in March before increasing again; the highest ratio was obtained in May (Figure 5).

**Figure 6:**
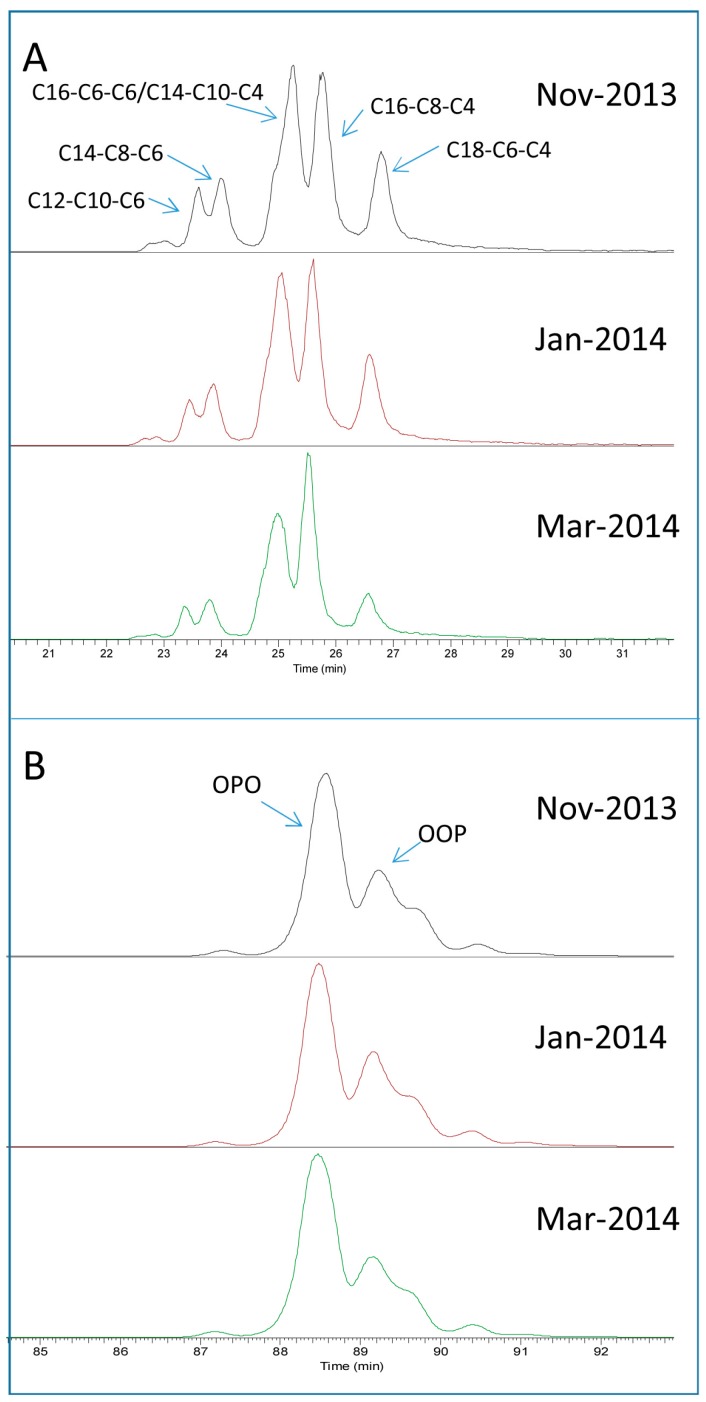
Isomer profile of two TAG groups across different seasons. (**A**) Isomeric species composition of TAG 28:0; (**B**) two positional isomers (OPO and OOP) of TAG 52:2.

In addition to the seasonal change of TAG profile at the group level, the proportion of different isomer species within the same group also fluctuated across seasons. Figure 6A shows the comparison of the relative proportion of the six isomer species of TAG 28:0 in November 2013 (spring), January 2014 (summer) and March 2014 (autumn); a clear increase of the proportion of TAG C4-C8-C16 and a concomitant decrease of that of TAG C4-C6-C18 was observed with the progression of the lactation. However, in the case of an abundant TAG group TAG 52:2, the proportion of the two positional isomers (OPO and OOP) did not change noticeably across these seasons, the OPO configuration being the dominant isomer regardless of seasons (Figure 6B). The MS/MS spectra of OPO and OOP from standards as well as from a milk sample are shown in (Figure S1, Supplementary Materials).

### 2.4. Inter-Cow Variation of TAG Profile

**Figure 7:**
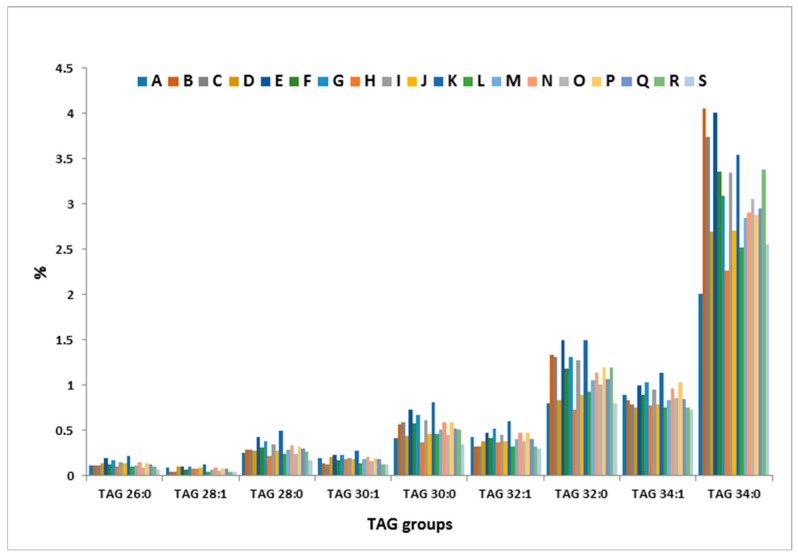
Inter-cow variation of some TAG groups (October 2013). Letters A–S denote the 19 cows.

Apart from the seasonal variation, a large cow-to-cow variation in TAG profile was also observed in this study. Figure 7 shows the variation in the proportion of nine TAG groups across the 19 animals measured in October 2013 as an example. It is interesting to note also that the inter-cow variation appears to be more pronounced with saturated TAG groups as compared to their mono-unsaturated analogues (Figure 7).

## 3. Discussion

Milk yield and composition change especially protein content and total fat content as influenced by various environmental and genetic factors have been extensively investigated over the last decades. Genomic regions associated with milk yield as well as protein and fat contents have been identified [19,20,21,22]. Regarding milk fat, most studies have focused either on total fat content or on fatty acid profile; few reports can be found on TAG profile, even though the latter is relevant to physical properties of fat, which affects both the processing and quality of dairy products. We have undertaken this study and compared the TAG profile over an entire milking season in South Victoria, Australia.

Over 100 triglyceride species have been identified in bovine milk in earlier studies [23,24]. A recent study provided a very detailed characterization of 243 TAG species in milk samples of different origins using NARP-LC/APCI-IT-TOF-MS [25]. However, complete separation and characterization of TAG isomers in bovine milk remains a challenge. Our recent survey revealed that at least 100 TAG groups can be found in milk samples without any requirement for enrichment. In this work, we have chosen 60 most abundant TAG groups for detailed quantification and comparison. Due to the lack of standards, only relative quantification was performed in this study, which is deemed adequate for comparative study and has been adopted widely in lipidomic analysis [26,27,28].

Although TAG is the major component of fat, not all TAG groups show the same level of correlation with total fat content. A number of groups especially TAG 36:1 and TAG 42:1 showed strong correlation with total fat content. It is interesting to note that these two TAG groups are not the most abundant groups in milk fat. In addition, a large number of strong correlations were identified across different TAG groups (Table S1, Supplementary Materials). Although it is known that TAG synthesis is not a random grouping of fatty acids, the underlying regulatory mechanism appears to be very complex, which requires further studies to fully understand.

TAG profile as judged by the proportion of different TAG groups is not constant over the milking season; most groups undergo a remarkable change and different seasonal patterns have been identified across the 60 groups. The overall unsaturation level of TAG has seen a substantial reduction in February/March and a boost in May, which may have important implication in the processing and properties of dairy products, given that the melting point and spreadability of fat is related to both the acyl chain length and the degree of unsaturation [29,30]. The clear seasonal variation of most TAG groups is an important finding, but the causative factors for such a variation remains unclear. Apart from climate variation, physiological and endocrinological changes associated with stage of lactation, and nutritional changes associated with pasture availability and supplementary feed inputs, may contribute to the availability of lipid precursors [31,32]. More studies using animals with consistent diets throughout the year may shed light on the biosynthesis regulation of TAG in the mammary gland. Irrespective of the cause, such a seasonal pattern is likely to be a general and reproducible feature for the region, since the experiment was conducted following the typical herd management regime of the region.

The most important finding of this study is probably the consistent proportion of positional isomers of TAG 52:2 across different seasons. TAG 52:2 contains two of the most abundant fatty acids of milk C16:0 and C18:1, the position of which in the glycerol backbone has attracted a considerable interest in recent years, due to the differential absorption efficiency by infants of C16:0 fatty acid at *sn*-2 and *sn*-1/3 position [14,15]. With the help of the standards, we could reveal the strict control of the ratio of positional isomer produced in the mammary gland for this particular TAG group. It remains to be determined whether this observation is applicable to other TAG groups.

Large variation in TAG profile was observed across different animals. Given that all the animals used in this experiment were at a similar stage of lactation and were under the same diet regime, this individual animal difference in TAG profile may be partly attributable to genetic variation across animals. Indeed, genetic correlation for the major fatty acids in bovine milk has been investigated in a number of studies [33,34], and heritability estimates were moderate to high for short- and medium-chain fatty acids and moderate for long-chain fatty acids [34]. More work is needed to understand the genetic determinism of the major TAG groups as well as their regiospecific structure.

## 4. Materials and Methods

### 4.1. Cows, Herd Management, and Milk Sample Collection

Milk samples were collected on a monthly basis for eight consecutive months (from October 2013 to May 2014) in Victoria (Australia) from 19 seasonally calving multiparous Holstein–Friesian dairy cows (labelled A–S), that calved in late winter/early spring. At the time of the first sample collection in October, they were 73 ± 24 days in milk. All cows were maintained in the research herd at the Department of Economic Development, Jobs, Transport and Resources’ Ellinbank Centre and the experimentation was conducted in accordance with the Australian Code of Practice for the Care and Use of Animals for Scientific Purposes [35]. Cow diet varied through the sampling period but the majority of the cows’ nutrient intake was usually derived from grazed pasture supplemented with bought in feedstuff fed according to different strategies. This included offering cereal grain or pelleted concentrates in the dairy at milking time and/or the provision of a mixed ration in a feedpad after milking. On each sampling occasion, the total milk from the afternoon and morning milking was collected into test buckets and pooled for each cow, and a subsample was taken for analysis. Milk samples were transported to the laboratory on ice and kept at −80 °C before analysis.

### 4.2. Chemicals

One TAG species (TAG tri-20:1) used as internal standard for TAG analysis, and two TAG positional isomer standards (TAG 18:1-16:0-18:1 and TAG 18:1-18:1-16:0) were purchased from Sigma-Aldrich (St. Louis, MO, USA). Solvents used for lipid extraction and mobile phase preparation were of chromatographic grade and were from Merck (methanol and acetonitrile) and Sigma-Aldrich (chloroform and isopropanol). Ammonium formate, used as mobile phase additive, was of analytical grade (Sigma-Aldrich).

### 4.3. Lipid Extraction from Milk

Extraction of lipids from raw milk was conducted based on the method of Folch et al. [36], with some modifications as reported previously [37]. To 0.2 mL of full cream milk, 0.8 mL of Milli-Q water was added, and the diluted milk was mixed with 4 mL of chloroform/MeOH (2:1, v/v) and shaken thoroughly for 10 min at room temperature. The mixture was then centrifuged for 10 min at 3000 rpm to facilitate phase separation. After transferring the organic phase to a new tube, the aqueous phase was extracted again with 2 mL of chloroform/MeOH (2:1, v/v). The combined organic phase (ca. 4.5 mL) was dried under a stream of nitrogen, and the samples were then reconstituted in isopropanol/chloroform (2:1) before LC-MS analysis.

### 4.4. TAG Analysis by LC-MS

Chromatographic separation for TAG identification was achieved using two Poroshell 120 EC-C18 columns (150 mm × 4.6 mm, 2.7 µm, Agilent Technologies, Santa Clara, CA, USA) connected in series on an Agilent 1290 Infinity HPLC system. The column compartment was maintained at 40 °C and the auto-sampler at 12 °C. The mobile phase was composed of acetonitrile/water (60:40, v/v) containing 10 mM ammonium formate (A) and acetonitrile/isopropanol (10:90, v/v) containing 10 mM ammonium formate (B). The flow rate was 0.5 mL/min with a gradient elution of 60 to 100% B over 90 min. The injection volume was 4 µL. The LC-MS profile of milk TAG after such a two-column separation is shown in Figure S2B (Supplementary Materials).

Chromatographic separation for TAG quantification was conducted using an Acquity UPLC HSS T3 column (100 mm × 2.1 mm, 1.8 µm, Waters, Dublin, Ireland) on the same Agilent HPLC system. The column compartment was maintained at 50 °C and the auto-sampler at 12 °C. The mobile phase was composed of acetonitrile/water (60:40, v/v) containing 10 mM ammonium formate (A) and acetonitrile/isopropanol (10:90, v/v) containing 10 mM ammonium formate (B). The flow rate was 0.28 mL/min with a gradient elution of 20 to 100% B over 20 min. The injection volume was 2 µL. The LC-MS profile of milk TAG after such a short separation is shown in Figure S2A (Supplementary Materials).

The detection of lipids was by LTQ-Orbitrap mass spectrometer (Thermo Scientific, Waltham, MA, USA) operated in electrospray ionization (ESI) positive Fourier transform mode. The heated capillary was maintained at 300 °C with a source heater temperature of 350 °C, and the sheath, auxiliary, and sweep gases were at 40, 15, and 5 units, respectively. The source voltage was set to 4.0 kV for positive mode. The resolution was set to 60,000. Identification of TAG groups and species present in milk was performed based on accurate mass of parent ions (± 5 ppm) and top5 MS/MS spectra (CE 35) acquired in IT mode. All TAG molecules were detected as their ammonium adducts in ESI mode and TAG ammonium adducts were used as precursors for MS/MS data acquisition; the fatty acid composition of TAG molecules was established based on DAG ions (characteristic product ions after neutral loss of one fatty acid chain). Selected TAG groups were quantified at a relative scale using peak area of parent ions after normalization by the internal standard.

### 4.5. Statistical Analysis of Data

All data analyses (correlation analysis and ANOVA) were performed using XLSTAT (Microsoft Excel, Version 2016.2); where significant differences were found between seasons, Tukey’s HSD test was conducted for multiple comparisons.

## 5. Conclusions

TAG profile of bovine milk is not constant over a milking season and the seasonal pattern varies with TAG groups. The most prominent change in TAG profile is a decrease in overall unsaturation level in late summer/early autumn and an increase in unsaturation level by the end of lactation (in late autumn). The proportion of isomeric species of different fatty acid composition within a same TAG group was also found to vary, but the positional isomer composition within a TAG group appears to be constant across different seasons.

## Supplementary Materials

The following are available online at www.mdpi.com/2218-1989/7/2/24/s1, Table S1: Pairwise correlation between different TAG groups and total fat content. Table S2: Statistical comparisons between the eight months for each TAG group. Figure S1: MS/MS spectra of OPO and OOP from standards and a milk sample. Figure S2: LC-MS profile (TIC) of milk TAG after one-column and two-column separation.
