# Supplementary material for: Seasonal Variation of Triacylglycerol Profile of Bovine Milk
Source: Metabolites. 2017 Jun 2;7(2):24. doi: 10.3390/metabo7020024 (PMC5487995; doi:10.3390/metabo7020024)
Supplement: Supplementary file 1 [file metabolites-07-00024-s001.zip › metabolites-188485-for publishing-supplementary/Fig. S1 & Fig. S2 (Supplementary Materials).pptx]

## Slide 1
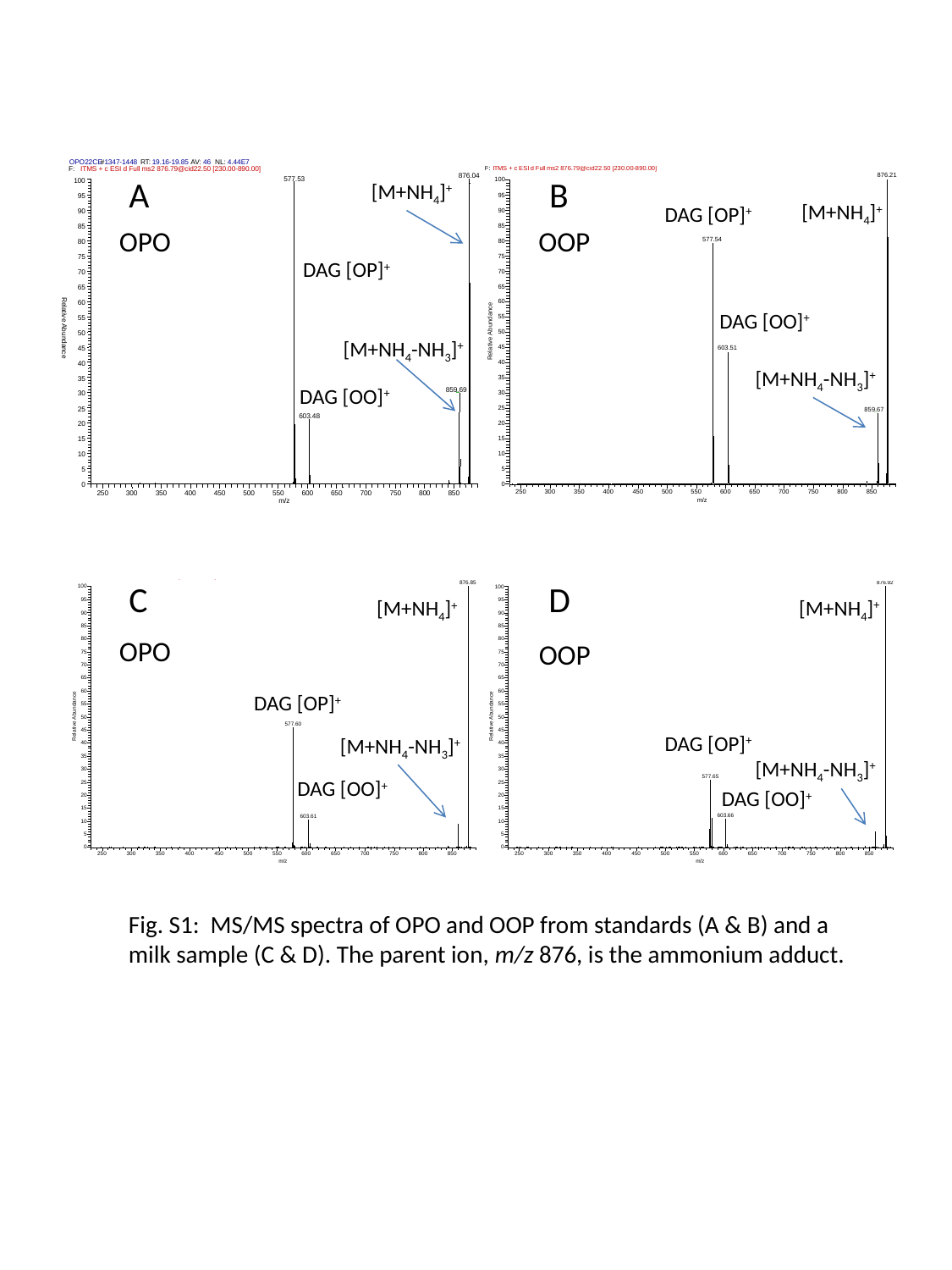

OPO22CE
#
1347-1448
RT:
19.16-19.85
AV:
46
NL:
4.44E7
F:
ITMS + c ESI d Full ms2 876.79@cid22.50 [230.00-890.00]
B
A
876.04
 [M+NH4]+
577.53
100
95
 [M+NH4]+
DAG [OP]+
90
OPO
OOP
85
80
DAG [OP]+
75
70
65
60
DAG [OO]+
55
Relative Abundance
50
 [M+NH4-NH3]+
45
40
 [M+NH4-NH3]+
35
DAG [OO]+
859.69
30
25
603.48
20
15
10
5
0
250
300
350
400
450
500
550
600
650
700
750
800
850
m/z
D
C
 [M+NH4]+
 [M+NH4]+
OPO
OOP
DAG [OP]+
DAG [OP]+
 [M+NH4-NH3]+
 [M+NH4-NH3]+
DAG [OO]+
DAG [OO]+
Fig. S1: MS/MS spectra of OPO and OOP from standards (A & B) and a milk sample (C & D). The parent ion, m/z 876, is the ammonium adduct.

## Slide 2
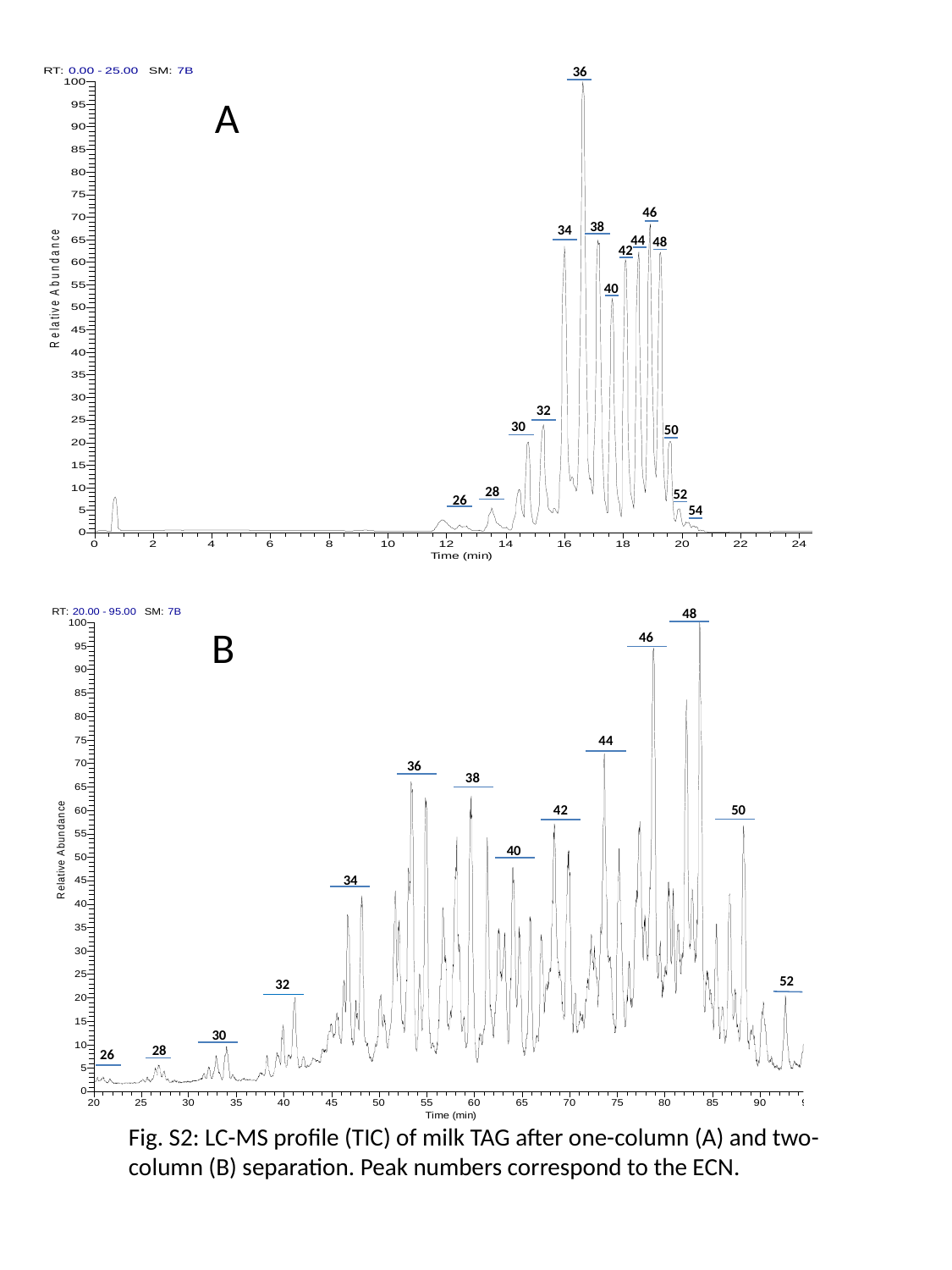

36
A
48
B
46
44
36
38
42
50
40
34
52
32
30
28
26
46
38
34
44
48
42
40
32
30
50
28
52
26
54
Fig. S2: LC-MS profile (TIC) of milk TAG after one-column (A) and two-column (B) separation. Peak numbers correspond to the ECN.
